# A human monoclonal antibody that specifically binds and inhibits the staphylococcal complement inhibitor protein SCIN

**DOI:** 10.1080/21505594.2017.1294297

**Published:** 2017-05-08

**Authors:** Hedzer Hoekstra, Francisco Romero Pastrana, Hendrik P. J. Bonarius, Kok P. M. van Kessel, Goffe S. Elsinga, Neeltje Kooi, Herman Groen, Jan Maarten van Dijl, Girbe Buist

**Affiliations:** a Department of Medical Microbiology, University of Groningen, University Medical Center Groningen, Groningen, The Netherlands; b IQ Therapeutics, Groningen, The Netherlands; c Medical Microbiology, University Medical Center Utrecht, Utrecht, The Netherlands

**Keywords:** C3b, complement, Monoclonal antibody, SCIN, *Staphylococcus aureus*

## Abstract

*Staphylococcus aureus* is a serious public health burden causing a wide variety of infections. Earlier detection of such infections could result in faster and more directed therapies that also prevent resistance development. Human monoclonal antibodies (humAbs) are promising tools for diagnosis and therapy owing to their relatively straightforward synthesis, long history of safe clinical use and high target specificity. Here we show that the humAb 6D4, which was obtained from a random screen of B-cells producing antibodies that bind to whole cells of *S. aureus*, targets the staphylococcal complement inhibitor (SCIN). The epitope recognized by 6D4 was localized to residues 26 to 36 in the N-terminus of SCIN, which overlap with the active site. Accordingly, 6D4 can inhibit SCIN activity as demonstrated through the analysis of C3b deposition on *S. aureus* cells and complement-induced lysis of rabbit erythrocytes. Importantly, while SCIN is generally regarded as a secreted virulence factor, 6D4 allowed detection of strongly increased SCIN binding to *S. aureus* cells upon exposure to human serum, relating to the known binding of SCIN to C3 convertases deposited on the staphylococcal cell surface. Lastly, we show that labeling of humAb 6D4 with a near-infrared fluorophore allows one-step detection of SCIN-producing *S. aureus* cells. Together, our findings show that the newly described humAb 6D4 specifically recognizes *S. aureus* SCIN, which can potentially be used for detection of human serum-incubated *S. aureus* strains expressing SCIN.

## Introduction

*Staphylococcus aureus* is a highly adaptable and dangerous Gram-positive bacterial pathogen that is asymptomatically carried by about one-third of the human population. *S. aureus* can cause a wide variety of infections due to its extensive arsenal of virulence factors.^1^ A subset of these virulence factors target the human immune system by blocking chemotaxis of phagocytes, complement activation, oxidative killing or phagocytic uptake. Alternatively, they may redirect host defenses, such as fibrin formation or formation of neutrophil extracellular traps to favor pathogen replication.^2^ Thus, the response of *S. aureus* to the human immune system is highly flexible, allowing survival in the host's hostile environment.^3^ Due to its adaptability *S. aureus* has also become resistant to a broad spectrum of antibiotics,^4^ and nowadays the drug-resistant lineages of *S. aureus* represent a serious public health burden.^2,5^ This applies in particular to methicillin-resistant *S. aureus* (MRSA), which causes significantly increased morbidity and mortality worldwide.^6,7^ Vancomycin has been the drug of choice to treat MRSA infections, but strains have emerged that display reduced vancomycin susceptibility.^8^ This implies that there is an urgent need for new and reliable approaches to prevent and treat infections by drug-resistant staphylococci.

Immune therapies against *S. aureus* infections have been explored as a treatment alternative to antibiotics. While active immunization could potentially prevent the onset of *S. aureus* infections, passive immunization could be applied to treat acute or current infections. While the use of pooled human sera does not seem to be very effective,^9,10^ passive immunization with monoclonal antibodies, preferably human monoclonal antibodies (humAbs), is an attractive alternative option. Importantly, humAbs have a high specificity, their synthesis is relatively straightforward, and they have a long history of safe use.^11,12^ However, despite recent successes in animal models,^13-15^ the efficacy of passive immunization with humAbs has not yet been confirmed in clinical trials.^11^

Wounds of patients with the genetic blistering disease epidermolysis bullosa (EB) are highly susceptible to bacterial colonization.^16^ In a study by van der Kooi-Pol *et al.*, it was documented that essentially all investigated EB patients with chronic wounds were heavily colonized with *S. aureus*.^17^ Interestingly, it was noted that these patients did not frequently suffer from *S. aureus* bacteraemia, despite the impaired barrier function of the skin. Compared to healthy individuals, the plasma of EB patients contained significantly higher IgG1 and IgG4 levels, suggesting a potentially protective effect of anti-staphylococcal antibodies against invasive staphylococcal infections.^18,19^ In a recent project, we therefore collected B-cells from donors with EB and applied them to develop of a set of fully human monoclonal antibodies against molecules exposed on the cell surface of *S. aureus*.^13-15^ The present study was aimed at the characterization of one of these humAbs referred to as 6D4. In brief, our results show that the humAb 6D4 binds specifically to the staphylococcal complement inhibitor (SCIN), thereby inhibiting its activity. Furthermore, using 6D4, we show that cell surface binding of SCIN is enhanced in the presence of human serum.

## Results

### Identification of a human monoclonal antibody that targets the staphylococcal complement inhibitor SCIN

**Figure 1.**
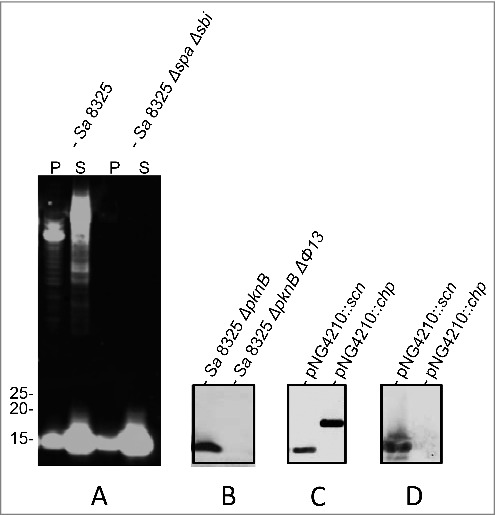
Identification of SCIN as target of humAb 6D4. Western blot analysis using humAb 6D4 on proteins from cell pellet (P) and growth medium fractions (supernatant; S) of the *S. aureus* (Sa) strains NCTC8325 and NCTC8325 Δ*spa*Δ*sbi* (A), and the growth medium fractions of strains NCTC8325 Δ*pknB* and NCTC8325 Δ*pknB* ΔΦ13 (B). Western blot analysis of the growth medium fractions of *L. lactis* pNG4210::*scn* or pNG4210::*chips* secreting the SCIN or CHIPS proteins, respectively, using anti-His-tag antibodies (C), or humAb 6D4 (D). Molecular weights (kDa) of marker proteins are indicated next to panel A.

The humAb 6D4 was identified from a random screen of B-cells producing antibodies that bind to whole cells of *S. aureus*. Consequently, the actual target of 6D4 was initially not known. To identify the antigen recognized by 6D4, immunoprecipitation experiments were performed. However, the subsequent Mass Spectrometric analysis of precipitated proteins yielded no conclusive identification of the respective antigen (not shown). As an alternative approach toward target identification, we performed a Western blotting analysis on cells and growth medium fractions of different *S. aureus* isolates. As expected, 6D4 bound to the immunoglobulin-binding proteins Spa (also known as protein A) and Sbi (Fig. 1A). In addition, 6D4 was found to bind a protein of 10–15 kDa that was present both in the cell and growth medium fractions of *S. aureus* NCTC8325, its derivative NCTC8325 (Δ*spa*Δ*sbi*) and NCTC8325 (Δ*pknB*) (Fig. 1, A and B). The respective signal was however absent from samples of *S. aureus* NCTC8325 (Δ*pknB*ΔΦ13) (Fig. 1B) and *S. aureus* SH1000 (not shown). The latter strains both lack the phage 13 (Φ13).^20^ This suggested that the antigen recognized by 6D4 was most likely an exported protein of 10–15 kDa encoded by Φ13. Indeed, Φ13 encodes 2 proteins, SCIN (13 kDa) and the Chemotaxis Inhibitory Protein of *S. aureus* (CHIPS; 17 kDa), which are known to be exported from the cytoplasm to the extracellular milieu.

To test whether 6D4 binds to SCIN or CHIPS, the respective genes were cloned and expressed with a His-tag in *Lactococcus lactis* strain PA1001. As shown by Western blotting with anti His-tag antibodies both SCIN and CHIPS were expressed and secreted by *L. lactis* upon induction with nisin (Fig. 1C). Importantly, the humAb 6D4 was found to bind specifically to SCIN (Fig. 1D). We considered this an important observation as SCIN is a potent inhibitor of the human complement system.^21-23^

### HumAb 6D4 binds to the active site of SCIN

**Figure 2.**
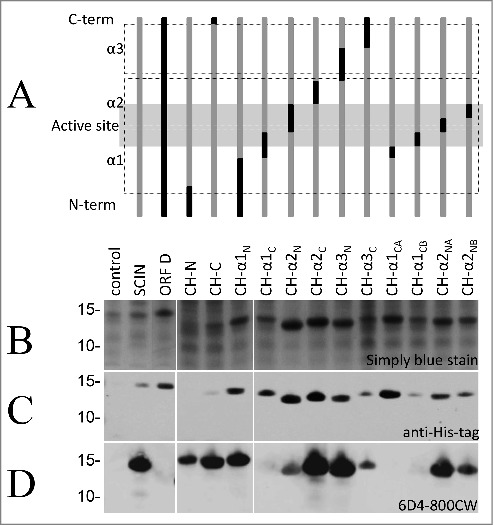
HumAb 6D4 binds to the C-terminal part of the first α-helix of SCIN. Proteins from *E. coli* Rosetta Gami expressing SCIN-OrfD chimera were separated using SDS-PAGE. The expressed chimera of SCIN and OrfD are schematically presented (A). The 3 helices (α1, α2 and α3) and the active site region of SCIN (in gray shading) are indicated. SCIN residues (gray) were exchanged with corresponding residues from OrfD (black). Exchanged residues (in parentheses) are: CH-N (1–13), CH-C (83–85), CH-α1_N_ (1–25), CH-α1_C_ (26–36), CH-α2_N_ (37–48), CH-α2_C_ (49–58), CH-α3_N_ (59–72), CH-α3_C_ (73–86), CH-α1_CA_ (26–30), CH-α1_CB_ (31–36), CH-α2_NA_ (37–42), and CH-α2_NB_ (43–48). Gels were stained with simply blue to verify protein production (B), and the produced proteins were specifically detected by immunoblotting with an anti-His-antibody (C) or the humAb 6D4–800CW (D). The positions of molecular weight marker proteins (kDa) are shown next to the gel and Western blot images.

To identify the specific SCIN epitope recognized by 6D4, we applied a set of previously constructed *Escherichia coli* Rosetta gami strains expressing IPTG-inducible His-tagged chimera of SCIN and its *S. aureus* homolog OrfD.^24^ The structure of these chimera is schematically represented in Fig. 2A, showing the relative positions of the 3 α-helices (α1, α2, and α3), the N- and C-termini, and the active site of SCIN. Of note, the OrfD protein has no identified biologic activity,^24^ and our humAb 6D4 does not bind to the full-size OrfD (Fig. 2D). All SCIN-OrfD fusion proteins were expressed upon IPTG induction, as shown by SDS-PAGE and Simply blue straining (Fig. 2B) or by immunodetection with anti-His tag antibodies (Fig. 2C), and all detected fusion proteins were of the expected size (Fig. 2, B and C). To assess the binding of 6D4 to the different SCIN-OrfD chimera, this humAb was labeled with the near-infrared fluorophore IRDye 800CW. In Western blotting analyses, the resulting 6D4–800CW facilitated the direct detection of SCIN at 800 nm equally well as the indirect detection of bound 6D4 with a secondary IRDye 800CW-labeled antibody at 800 nm (results not shown). As shown in Fig. 2D, bound to most SCIN-OrfD chimera. However, the 6D4–800CW did not bind the CH-α1-CA fusion, while the CH-α1-C and CH-α1-CB fusions were barely bound (Fig. 2D). These findings imply that the epitope recognized by 6D4 is located within the C-terminal half of the first α-helix of the SCIN protein, within amino acid residues 26 to 36. Importantly, these residues overlap with the active site of the SCIN protein.^24^

### HumAb 6D4 specifically binds the *S. aureus* SCIN protein

**Figure 3.**
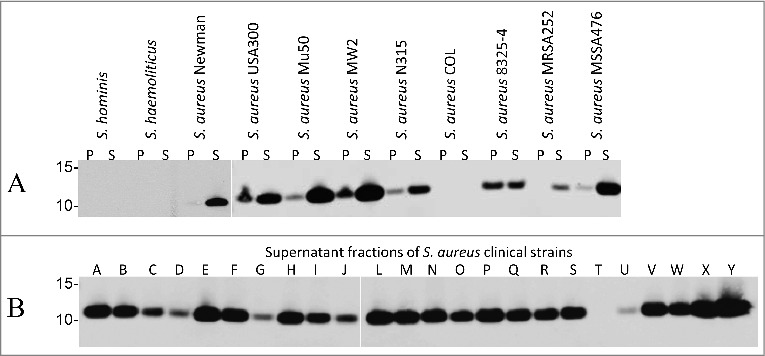
Binding of the humAb 6D4 to SCIN produced by different laboratory strains and clinical isolates of *S. aureus*. Western blotting analysis using humAb 6D4–800CW to detect SCIN in the cell pellet (P) or growth medium (S) fractions of *S. hominis, S. haemolyticus* and the *S. aureus* strains Newman, USA300, Mu50, MW2, N315, COL, NCTC8325–4, MRSA252 and MSSA476 (A), or in the growth medium fractions (supernatant) of 24 clinical *S. aureus* isolates named A-J and L-Y (B). Molecular weights (kDa) of marker proteins are indicated to the left of panels A and B. Loading of comparable amounts of proteins was confirmed by Simply Blue staining (not shown).

To verify the specificity of 6D4 for *S. aureus*, we performed a BLAST analysis using the NCBI protein database to identify other bacteria containing SCIN-encoding genes. This showed that the presence of SCIN was restricted to *S. aureus*, and that proteins with limited sequence similarity to SCIN were encoded by the genomes of only few other *Staphylococcus* species, including *S. argenteus* (61% identity from 89% query cover, GenBank: CDR22445.1), *S. hominis* (53% identity from 73% query cover, GenBank: EEK11996.1) and *S. haemolyticus* (57% identity from 74% query cover, GenBank: CPM70056.1). In none of these SCIN homologues was the epitope recognized by 6D4 (i.e. residues 26 to 36) fully conserved. This was confirmed by Western blotting analyses, where 6D4–800CW showed no binding to proteins from *S. hominis* or *S. haemolyticus*, while clear binding to the SCIN proteins of different sequenced *S. aureus* strains was detected (Fig. 3A). Of note, our BLAST analysis indicated that *S. aureus* COL does not contain the *scn* gene encoding SCIN and, consistent with this finding, 6D4–800CW did not bind to any protein of *S. aureus* COL (Fig. 3A).

### SCIN is detectable in most clinical *S. aureus* isolates

To explore the production of SCIN by clinical isolates of *S. aureus*, this was assessed with 6D4–800CW in a set of 24 clinical *S. aureus* isolates from the University Medical Center Groningen of which 22 were previously shown by PCR to carry the *scn* gene.^25^ Intriguingly, Western blotting with 6D4–800CW revealed the presence of SCIN in 23 of the 24 tested isolates (Fig. 3B), including isolate G which had tested negative for *scn* in the previous PCR analysis. In contrast, isolate T which had also tested negative for *scn* in the previous PCR also tested negative in the Western blotting with 6D4–800CW. A renewed PCR using *scn*-specific primers showed that the *scn* gene was indeed present in isolate G (data not shown), which is consistent with the detection of SCIN with 6D4–800CW in this isolate. Altogether, these results show that humAb 6D4 labeled with IRDye 800CW can be applied for the specific identification of clinical *S. aureus* isolates expressing SCIN.

### Serum incubation increases binding of SCIN to *S. aureus* cells

The *S. aureus* SCIN protein specifically inhibits the human complement system, one of the most important components of the innate immune system.^24,26-29^ This is achieved through the binding of SCIN to the C3b moiety of human C3 convertases on the bacterial surface, leading to their stabilization in a catalytically inactive form and preventing enhanced conversion of C3 into C3b as part of the so-called ‘alternative pathway’ in innate immunity. In addition, SCIN promotes the formation of inactive convertase dimers that preclude C3b binding by the complement receptor of phagocytic cells.^27,30^ Because the C3 convertases are key initiators in the complement activation cascades, effector functions such as C3b-mediated phagocytosis and C5a-mediated cell recruitment are effectively prevented by SCIN.^21,23,24,27-30^

**Figure 4.**
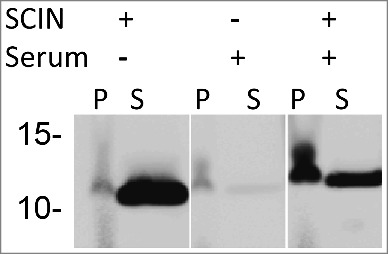
Binding of SCIN to *S. aureus* cells increases upon incubation in serum. Western blotting analysis of *S. aureus* Newman Δ*spa*Δ*sbi* cells collected by centrifugation (P) and growth medium fractions (S) using 6D4–800CW. The presence or absence of C3 convertases due to serum incubation, and the addition or absence of SCIN are indicated with + or -, respectively.

From the Western blotting analyses shown in Figs. 1 and 3, it was evident that SCIN is mostly detectable in growth medium fractions, and only to minor extent in the cell fractions when cells are grown in Tryptic Soy Broth (TSB). The latter is consistent with the previously documented finding that SCIN binds to the C3 convertases, which are formed on the *S. aureus* cell wall after initial C3b deposition.^26^ Therefore, we hypothesized that SCIN is likely more abundant in the cell fraction when cell wall-attached C3b is present. To verify this idea, *S. aureus* Newman Δ*spa*Δ*sbi* cells were covered with C3b through incubation in human sera and, subsequently, these cells were incubated in the presence or absence of added SCIN. As reflected by 6D4–800CW binding upon Western blotting, cells not incubated in serum displayed low levels of SCIN, whereas the respective supernatant fractions yielded a high signal due to the presence of SCIN (Fig. 4). Similarly, the serum-incubated samples without added SCIN showed a low signal in both the cell- and the respective supernatant fractions. In contrast, the serum-incubated samples with added SCIN showed a high SCIN-specific signal in the cell fraction and a lowered signal in the supernatant fraction (Fig. 4). These results show that the enhanced SCIN binding to the *S. aureus* cell wall due to the deposition of C3b and C3 convertases is readily detectable with the 6D4–800CW humAb.

**Figure 5.**
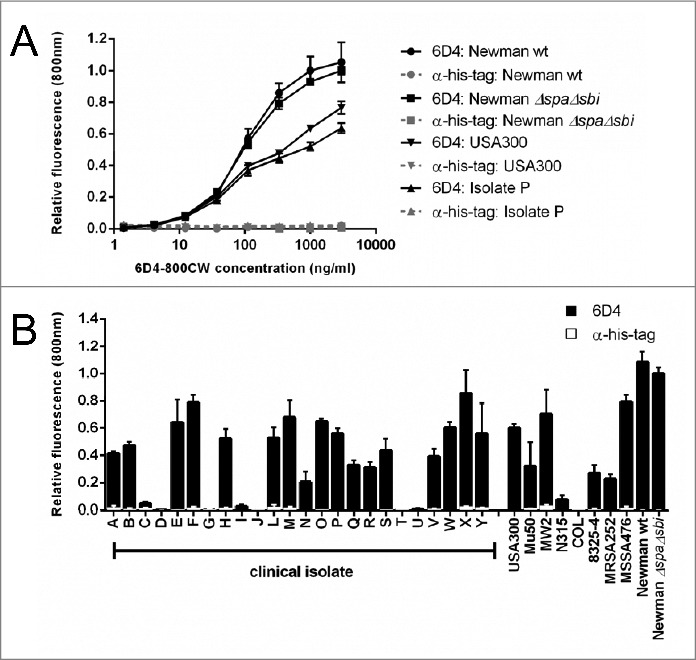
Binding of the humAb 6D4 to whole cells of *S. aureus*. Plates were coated with whole cells of various *S. aureus* clinical isolates or laboratory strains harvested from cultures in the mid-exponential growth phase where the growth medium was supplemented with human serum. 6D4–800CW was used for the detection of cell-bound SCIN, and an α-his-tag antibody was used as a negative control. Fluorescence readings at 800 nm are plotted relative to the binding of 6D4–800CW to *S. aureus* Newman *ΔspaΔsbi*. All measurements were performed in triplicate and the mean ± standard error (error bars) is shown. (A) concentration-dependent binding of 6D4–800CW to *S. aureus* Newman *ΔspaΔsbi*, Newman wild-type (wt), the clinical *S. aureus* isolate P, or the MRSA strain USA300 is indicated in in black symbols; the lack of binding of the α-his-tag control antibody to *S. aureus* Newman *ΔspaΔsbi*, Newman wild-type (wt), isolate P, or USA300 is shown in gray symbols. (B) Binding of 6D4–800CW to *S. aureus* Newman *ΔspaΔsbi*, various clinical *S. aureus* isolates and the sequenced *S. aureus* strains USA300, Mu50, MW2, N315, COL, 8325–4, MRSA252, MSSA476, Newman wild-type (WT) and Newman *ΔspaΔsbi* is indicated with black bars; binding of 100 ng/mL isotype control antibody IQNPA to the *S. aureus* clinical isolates and sequenced *S. aureus* strains as specified is indicated with white bars.

A plate assay was used to assess whether whole *S. aureus* cells could be detected after incubation with human sera using 6D4–800CW. Indeed, 6D4–800CW was found to bind concentration-dependently to the *S. aureus* clinical isolate P, and the strains USA300, Newman wild-type and Newman *ΔspaΔsbi* (Fig. 5A). In this assay binding of 6D4 to Spa and Sbi via the Fc-region was blocked by the addition of unrelated rabbit IgG, and effective blocking was confirmed with a control His-tag-specific rabbit antibody (α-his-tag; Fig. 5A). Importantly, 6D4–800CW allowed the detection of cell-bound SCIN in 19 of 24 clinical *S. aureus* isolates tested (Fig. 5B) Here it is noteworthy that 5 isolates showed no enhanced binding of SCIN, including 4 *scn*-proficient isolates and the isolate T lacking the *scn* gene. Furthermore, 6D4–800CW allowed detection of cell-bound SCIN for 8 of 9 sequenced *S. aureus* strains, where only the COL strain that lacks the *scn* gene yielded no signal (Fig. 5B). Binding of the α-his-tag control antibody was low for all strains due to blocking with an unrelated rabbit IgG (Fig. 5B).

### Direct detection of SCIN bound to the surface of *S. aureus* cells

**Figure 6.**
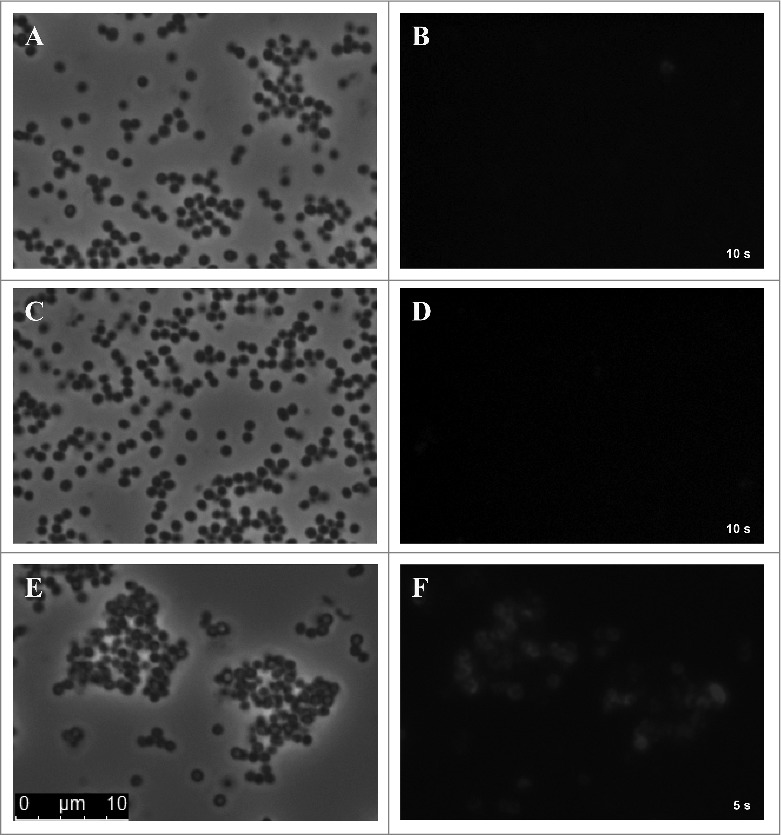
Serum-incubated *S. aureus* cells display elevated levels of SCIN binding. Phase contrast (panels A, C, E) and subsequent fluorescence microscopy at 800 nm (panels B, D, F) of cells of *S. aureus* Newman Δ*spa*Δ*sbi* collected from an overnight culture. Specifically, the panels show cells from the overnight culture (A, B), cells treated with serum but without the addition of SCIN (C, D), and cells treated with serum and added SCIN (E, F). Cell-bound SCIN was detected using 6D4–800CW.

For direct detection of SCIN bound to the surface of *S. aureus* cells, samples of *S. aureus* Newman Δ*spa*Δ*sbi* were prepared and spotted onto glass slides for fluorescence microscopy at 800 nm. *S. aureus* cells grown under standard culturing conditions and incubated with 6D4–800CW displayed almost no fluorescence and individual cells could not be distinguished (Fig. 6, A and B). Further, cells incubated in serum, but lacking added SCIN, showed no fluorescent signal at all (Fig. 6, C and D). Importantly however, serum-incubated cells with added SCIN showed a strongly enhanced fluorescent signal at 800 nm (Fig. 6, E and F). Here individual cells were detectable, though it is noteworthy that not all cells appeared to be fluorescently tagged. Taken together, these observations show that *S. aureus* cells incubated with human serum have a high potency for binding of SCIN, most likely due to the deposition of C3b and C3 convertases, which can be detected with IRDye 800CW-labeled 6D4 humAb.

### Impact of 6D4 on SCIN activity

**Figure 7.**
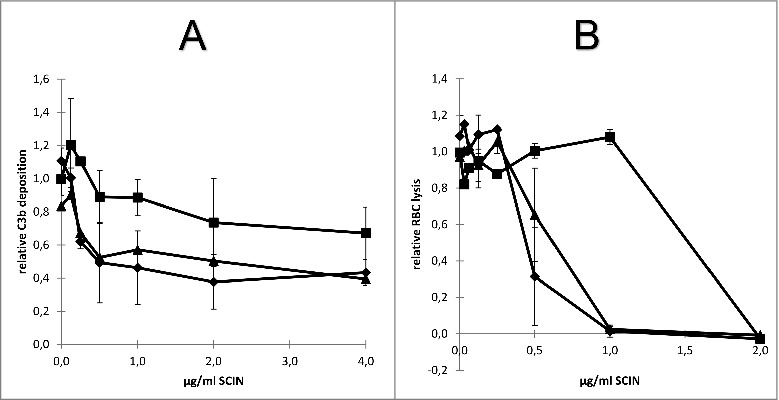
Impact of humAb 6D4 on SCIN activity. (A) C3b deposition on *S. aureus* Newman Δ*spa*Δ*sbi* cells upon preincubation of SCIN with humAb 6D4 (■). C3b deposition was monitored by flow cytometry. As a negative control, SCIN was preincubated with buffer (♦), or control IgG (▴). Each data point represents the mean ± standard error (error bars) of 3 independent experiments. (B) Reduced SCIN-mediated protection of rabbit erythrocytes against lysis by complement upon incubation of SCIN with humAb 6D4 (■). Hemolysis was quantified by pelleting of erythrocytes and subsequent measurement of the absorbance of supernatants at 450 nm. As a control, SCIN was preincubated with buffer (♦), or control IgG (▴). Each data point represents the mean ± standard error (error bars) of 2 separate experiments.

Since the humAb 6D4 binds to the active site of SCIN, we asked the question how this antibody impacts on the deposition of C3b on the *S. aureus* cell surface. To this end, we used an essay where increasing amounts of SCIN were pretreated with 6D4, before mixing with human serum. As controls, the SCIN protein was mock-treated with buffer or a control IgG before mixing with serum. Next, *S. aureus* Newman Δ*spa*Δ*sbi* cells were incubated for 30 min with the serum containing SCIN (with or without 6D4 pretreatment), after which the presence of C3b on the staphylococcal cell surface was measured by flow cytometry. As shown in Fig. 7A, in this assay the preincubation of SCIN with humAb 6D4 resulted in a relative deposition of C3b on the *S. aureus* cells close to 1, which represents the maximal C3b deposition upon incubation with serum. In contrast, the C3b deposition was inhibited by SCIN in the absence of 6D4. These findings imply that 6D4 can interfere with the deposition of C3b on the *S. aureus* cells.

An alternative possibility to measure the impact of 6D4 on SCIN activity is provided by the fact that complement causes the lysis of rabbit erythrocytes, and that this hemolysis can be inhibited by SCIN. To assess whether SCIN-mediated inhibition of the alternative pathway's hemolytic activity can be suppressed by 6D4, we pre-treated increasing amounts of SCIN with 6D4, before mixing with human serum and erythrocytes. As a negative control, the SCIN protein was either mock-treated with buffer or a control IgG before mixing with serum and erythrocytes. Next, the erythrocytes were incubated for 60 min with the human serum containing SCIN (with or without 6D4 pre-treatment), after which the erythrocytes were pelleted and the absorbance of supernatants at 450 nm was measured to assess the erythrocyte lysis. As shown in Fig. 7B, the preincubation of SCIN with 6D4 significantly reduced the protective effect of SCIN with respect to erythrocyte lysis, as compared with SCIN preincubated with the control IgG or with buffer. These observations fully support the view that the activity of SCIN can be inhibited by the humAb 6D4.

## Discussion

In this study, we show that the humAb 6D4 binds to the first α-helix of the staphylococcal complement inhibitor SCIN, which covers part of this protein's active site domain. Consistent with this finding, 6D4 interferes with the activity of SCIN, as shown through the analysis of C3b deposition on *S. aureus* cells and suppression of the protective effect of SCIN in the alternative pathway-mediated hemolysis of rabbit erythrocytes. Furthermore, we show that 6D4 labeled with the near-infrared fluorophore IRDye 800CW can be readily used to visualize the production and subcellular localization of SCIN by *S. aureus*.

The analysis of publicly available bacterial genome sequences suggests that the *scn* gene is specific for *S. aureus* isolates causing infections in humans. While sequenced *S. hominis* and *S. haemolyticus* strains contain genes with some sequence similarity to the *S. aureus scn* gene, the tested *S. hominis* and *S. haemolyticus* strains did not bind humAb 6D4. This underpins the conclusion that this humAb is highly specific for *S. aureus* SCIN, and suggests that it will bind preferentially to isolates associated with infections in humans. Previous studies have reported that SCIN may be present in 90% of all clinical *S. aureus* isolates and that it is expressed *in vivo*.^21-24^ Consistent with this view, we observed that, from a panel of 33 tested *S. aureus* isolates, only 2 did not express SCIN.

SCIN is a potent antigen that evokes high antibody titres in *S. aureus*-colonized individuals.^18,31,32^ Under the *in vitro* conditions used for culturing *S. aureus* in this study, the clearest SCIN signals were obtained for growth medium fractions, while the signals in the respective *S. aureus* cell fractions were relatively low. On the other hand, our present findings show that SCIN was effectively recruited to the *S. aureus* cell surface when this bacterium was exposed to human serum. This phenomenon was also clearly evident at the single cell level by fluorescence microscopy. The observed redistribution of SCIN is consistent with the fact that SCIN binds to the C3b moiety of C3 convertases upon their deposition on the bacterial cell surface.^33^ This puts emphasis on the extensive interactions between *S. aureus* and its human host, which are underestimated under the generally applied *in vitro* culturing conditions. Indeed this view is confirmed by a previous study showing that *S. aureus* cells bind a variety of human proteins to their cell surface upon incubation in plasma.^34^ Of note, when serum-incubated clinical *S. aureus* isolates and laboratory strains were tested for enhanced binding of SCIN using 6D4–800CW, only 4 out of 34 investigated strains remained undetectable, which suggests that they only bind small amounts of SCIN. Notably, our Western blotting analyses show that these strains produce relatively low amounts of SCIN, which might not be sufficient to distinguish the SCIN-specific signal from the background signal in a whole cell plate reader-based approach. Of note, upon fluorescence microscopy, not all S. aureus cells appeared to bind equal amounts of 6D4–800CW, suggesting that there may be cell-to-cell differences in the formation of C3 convertases, the binding of SCIN or the binding of 6D4–800CW.

In conclusion, in the present study we present a humAb that binds to the active site of the *S. aureus* SCIN protein, especially residues 26-36. While the humAb 6D4 does interfere with the activity of SCIN, it seems rather unlikely that it can be applied in antistaphylococcal therapy since SCIN-deficient variants of *S. aureus* can also cause infections. Importantly however, the IRDye 800CW-labeled version of this humAb (i.e., 6D4–800CW) can be applied to specifically detect *S. aureus* isolates that express SCIN, an important virulence factor that allows *S. aureus* to effectively evade the human complement system. A completely novel finding is that SCIN binding to the staphylococcal cell-surface is substantially enhanced in the presence of human serum. Since SCIN production is associated in particular with *S. aureus* isolates that caused infections in humans, our SCIN-specific antibody may find potential future applications in the identification of *S. aureus* lineages with a high potential for causing infections. This could not only involve diagnostic tests, but also *in vivo* imaging approaches for which proof-of-principle was recently obtained using vancomycin labeled with the IRDye 800CW.^35-37^

## Materials and methods

### Strains and growth conditions

Strains used in this study are listed in Table 1. *E. coli* Rosetta Gami (DE3) pLysS strains (Novagen, Merck Biosciences Darmstadt, Germany) carrying prSETB-derived plasmids with the genes encoding for SCIN, OrfD or the respective chimeric constructs have been described previously.^24^ *E. coli* Rosetta gami strains were grown overnight in Lysogeny Broth (LB, Becton Dickinson, Breda, The Netherlands) at 37°C under vigorous agitation (250 rpm), in the presence of ampicillin (50 μg/ml) and chloramphenicol (34 μg/ml) for plasmid selection. All staphylococcal strains were cultured overnight in TSB (Oxoid Limited, Hampshire, UK) at 37°C under vigorous agitation (250 rpm), unless otherwise specified. *L. lactis* strains were grown at 30°C in M17 broth (Oxoid Limited), or on plates containing 1.5% agar and 0.5% glucose (wt/vol), supplemented with chloramphenicol (5 µg/ml) for plasmid selection.

**Table 1.** Strains and plasmids used in this study.

Strains	Relevant phenotype(s) or genotype(s)	Reference or Source
*S. aureus* Newman	NCTC 8178 clinical isolate	^40^
*S. aureus* Newman Δ*spa*Δ*sbi*	*spa sbi* mutant	^41^
*S. aureus* USA300	Community-acquired MRSA isolate	^42^
*S. aureus* SH1000 Δ*spa::kan*	*rsbU^+^, agr^+^*; replacement of *spa* by kanamycin resistance marker (Kan^R^)	^1^
*S. aureus* N315	Hospital-acquired MRSA isolate	^43^
*S. aureus* NCTC8325 Δ*pknB*	NCTC8325 (wild-type, 11-bp deletion in *rsbU*) containing *pknB* deletion	^44^
*S. aureus* NCTC8325 Δ*pknB* ΔΦ13	NCTC8325 *ΔpknB* that had lost the phage 13	^45^
*S. aureus* NCTC8325 Δ*spa*Δ*sbi*	*spa sbi* mutant	^42^
*S. aureus* NCTC8325–4	Prophage cured and restriction-deficient derivative of NCTC 8325	^46^
*S. aureus* Mu50	Hospital-acquired vancomycin resistant isolate	^43^
*S. aureus* MW2	Community-acquired MRSA isolate	^47^
*S. aureus* COL	Early hospital-acquired MRSA isolate	^48^
*S. aureus* MRSA252	Hospital-acquired MRSA isolate	^49^
*S. aureus* MSSA476	Community-acquired methicillin sensitive isolate	^49^
*S. aureus* isolates A-J and L-Y	Community- and hospital-acquired clinical isolates collected during a 4.5-year period in the UMCG from 19 patients with different clinical symptoms (for detailed strain descriptions see reference)	^25^
*S. haemolyticus*	Opportunistic pathogen clinical strain from UMCG	This study
*S. hominis*	Human commensal strain obtained from UMCG	This study
*E. coli* Rosetta gami (DE3) pLysS	DE3 lysogen contains T7 polymerase upon IPTG induction.	(Novagen)
*L. lactis* PA1001	MG1363 *pepN*::*nisRK*, Δ*acmA* Δ*htrA*	^50^
**Plasmids**		
pNG4210	Cm^R^, containing P*_nisA_*, SS*_usp45_, Bam*HI/*EcoR*I-*Xba*I/*Not*I cloning sites, and *his_6_*	^38^
pNG4210::*scn*	pNG4210 containing *scn* with C-terminal *his_6_*	This study
pNG4210::*chp*	pNG4210 containing *chp* with C-terminal *his_6_*	This study
prSETB::*scn*/*orfD*	Vectors for expression of chimeric SCIN/OrfD fusions	^24^

*Notes*. Cm^R^, chloramphenicol resistance gene; *P_T7_*, IPTG inducible T7-promoter; *P_nisA_*, nisin-inducible promoter; *his_6_*, 6x histidine tag; *SS_usp45_*, signal sequence of *usp45*; *MCS*, multiple cloning site

### Sample preparation, SDS/LDS-PAGE, western blotting and immunodetection

For the production of chimera of SCIN and the homologous OrfD protein of unknown function overnight cultures of described previously *E. coli* Rosetta gami strains^24^ were diluted to an optical density at 600nm (OD_600_) of 0.1. Chimeric protein production was induced at an OD_600_ of ∼0.5 by the addition of 1 mM isopropyl-β-D-thiogalactopyranoside (IPTG). After 4 h of continued cultivation, cells were collected by centrifugation, and the SCIN-OrfD chimeras produced by these cells were separated by SDS-PAGE as described previously.^24^ The replacement of SCIN residues with corresponding OrfD residues is detailed in Fig. 2 and the corresponding legend.

For the preparation of LDS-PAGE samples, *S. aureus* cells collected by centrifugation were disrupted with 0.1 µm glass beads (Biospec Products, Bartlesville, USA) in a Precellys 24 homogenizer (Bertin Technologies, France), and resuspended in LDS sample buffer (Life Technologies). Growth medium fractions were prepared for LDS-PAGE as described before^1^ Proteins were separated on NuPAGE gels (Life Technologies) and either visualized by Simply Blue Safe Staining (Life Technologies)^1^ or Western blotting using either mouse anti-His tag (Life Technologies), IRDye 800CW-labeled humAb 6D4, or IRDye 800CW-labeled secondary goat anti-human or goat anti-mouse antibodies (LI-COR Biosciences). Bound antibodies were visualized using an Odyssey Infrared Imaging System (LI-COR Biosciences).

### Expression of staphylococcal SCIN and CHIPS proteins in *L. lactis*

Primers used for cloning are described in Table 2. DNA amplification was performed using Fusion Hot start High-Fidelity DNA polymerase according the instructions of the supplier (Thermoscientific). Bacterial chromosomal DNA was isolated using the ZR BAC DNA Miniprep Kit (Zymo Reasearch Corporation, USA) following the manufacturer's protocol. Primer pairs Scin-up/Scin-low used for detection of *scn*, the gene encoding SCIN, were used as described previously.^25^ Cloning of the PCR-amplified *scn* and *chp* genes was performed by *Not*1 and *Bam*H1 (New England Biolabs) cleavage followed by ligation to *Not*I/*Bam*HI cleft plasmid pNG4210.^38^ Ligated mixtures were used to transform electrocompetent *L. lactis* PA1001 as described.^39^ All constructs thus obtained were verified by sequencing (Eurofins MWG Operon, Ebersberg, Germany).

**Table 2.** Primers used for detection or cloning of *scn* and *chp* genes.

Primer	Sequence 5′>3′	Enzyme^*^
Scn F	ATATGGATCCACAAGCTTGCCAACATCGAATGAATATC	*Bam*HI
Scn R	ATATGCGGCCGCATATTTACTTTTTAGTGCTTCGTCAATTTC	*Not*I
Chp F	ATATGGATCCTTTACTTTTGAACCGTTTCCTACAAATG	*Bam*HI
Chp R	ATATGCGGCCGCGTATGCATATTCATTAGTTTTTC	*Not*I
Scin-up	AGTCTTTTGACTTAAGAGC	
Scin-low	GTTTTAGCATCACCACTAGTA	

*Notes*.

* , restriction enzyme sites are underlined in the nucleotide sequences.

The production of secreted SCIN and CHIPS in exponentially growing (∼0.5 OD_600_) cultures of *L. lactis* was induced by the addition of nisin (3 ng/ml, Sigma-Aldrich, St. Luis, MO). Growth medium fractions were harvested after overnight incubation at 30°C, and proteins in these fractions were analyzed by LDS-PAGE, Simply Blue Safe Staining, or Western blotting as described above.

### *S. aureus* incubation in human sera

Cells of *S. aureus* Newman Δ*spa*Δ*sbi* were collected from the growth medium by centrifugation at 14.000 rpm for 2 min. The supernatant fraction, containing secreted SCIN, was collected. Next, the collected cells were resuspended and incubated with 20% human serum in HBS (Hepes Buffered Saline; 20mM Hepes, 140 mM NaCl) *plus* 5 mM CaCl_2_ and 2.5 mM MgCl_2_ for 30 min to coat the bacteria with C3B and allow for the formation of C3 convertases. Subsequently, the cells were incubated in PBS at 37°C for 30 min to dissociate surface-bound C2a/Bb. Where appropriate, the collected *S. aureus* supernatant was added to the C3 convertase-covered bacteria to allow binding of SCIN to the surface-attached C3 convertase. The protocol for blood donations from healthy volunteers was approved by the Independent Ethics Committee of the Foundation ‘Evaluation of Ethics in Biomedical Research’ (Assen, the Netherlands). This protocol is registered by QPS Groningen (code 04132-CS011). The required written consent was obtained for all donors included in the present studies.

### Detection of SCIN bound to whole cells of *S. aureus*

*S. aureus* isolates were grown overnight in TSB, diluted 1:100 in fresh medium and cultured until the mid-exponential growth phase (OD_600_ ∼0.5). Next, the cells were coated with complement by adding serum (end concentration 20%) and incubation was continued for 30 min. After this incubation, the bacteria were washed with phosphate-buffered saline (PBS). High-binding ELISA plates for fluorescence measurements (Greiner Bio-one) were coated with 5 × 10^6^ colony forming units (CFU) per well in PBS for 18 h at 4°C. Plates were blocked with 4% BSA in PBS with 0.05% Tween-20 (PBST). Surface-bound IgG Fc-binding proteins of *S. aureus* (i.e., Spa and Sbi) were saturated with 100 μg/mL normal rabbit immunoglobulin fraction (DAKO) in PBST containing 1% BSA. The humAb 6D4 was labeled with IRDye 800CW (LI-COR Biosciences, Bad Homburg, Germany) by incubation for 2 hours with 20 µg of IRDye 800CW per mg of protein in PBS (pH 8.5). The mix was desalted following the manufacturer's instructions with a PD minitrap G-25 desalting column (GE Healthcare, Germany). The resulting 6D4–800CW was stored in the dark at 4°C. To quantify the binding of 6D4–800CW to serum-incubated whole cells with added SCIN, the plates were incubated with 300 ng/mL 6D4–800CW in PBS for 30 min, washed thrice with PBS and scanned with the Odyssey infrared imaging system (Li-Cor Biosciences) for fluorescence at 800 nm.

### Fluorescence microscopy

Overnight cultures in TSB were diluted to an OD_600_ of 10. Untreated samples were taken from the overnight culture. Convertase-covered cell samples were obtained as described above. Cells were collected by centrifugation at 14.000 rpm for 2 min and washed with PBS. The washed cells were incubated with the 6D4–800CW (3000 ng/mL in PBS) for 30 min. After the incubation, the cells were collected by centrifugation at 14,000 rpm for 2 min and washed with PBS. Next, cells were spotted on a glass slide for microscopy, and a coverslip was mounted and sealed. Fluorescence microscopy was performed using a Leica DM5500B epifluorescence microscope equipped with an 800 nm filter block. Images were captured with a Leica DFC365FX camera using a 63x objective (Leica Microsystems BV, The Netherlands).

### Determination of C3b deposition on *S. aureus* cells

Cells of *S. aureus* Newman Δ*spa*Δ*sbi* were collected as described above, and 5×10^7^ CFU/ml were incubated with 5% pooled normal human serum in HBS *plus* 5 mM CaCl_2_, 2.5 mM MgCl_2_ and 0.1% human serum albumin for 30 min at 37°C while shaken at 700 rpm. Different concentrations SCIN (0–4 µg/ml) were preincubated with the purified humAb 6D4 (10 µg/ml), with the control human anti-DNP IgG1 (10 µg/ml, Genmab, Utrecht), or with HBS buffer for 10 min at room temperature before mixing with the serum. Bacteria were washed by centrifugation and incubated with 1 µg/ml anti-C3b mAb (Quidel Corp.) for 30 min at 4°C followed by APC-labeled Goat-anti-Mouse-Ig (BD Biosciences). Samples were fixed with 1% paraformaldehyde (Polysciences) and analyzed on a FACSVerse flow cytometer (BD Biosciences). Data are expressed relative to the mean fluorescence value of bacteria incubated in serum only.^23^

### The alternative pathway hemolytic assay

Washed rabbit erythrocytes at 1×10^8^ c/ml (Biotrading) were incubated with 5% pooled normal human serum in HBS *plus* 10 mM MgCl_2_ and 10 mM EGTA for 60 min at 37°C while shaken at 600 rpm. Different concentrations SCIN were preincubated with purified humAb 6D4 (10 µg/ml), with the control human anti-DNP IgG1 (10 µg/ml, Genmab, Utrecht), or with HBS-buffer *plus* 10 mM MgCl_2_ and 10 mM EGTA for 10 min at room temperature before mixing with serum. Erythrocytes were pelleted and the absorbance of supernatants at 450 nm was measured. Data are expressed relative to the mean value measured for erythrocytes incubated with serum only, which was set to 1.^21^
